# Propensity score matched analysis for the safety and effectiveness of remdesivir in COVID-19 patients with renal impairment

**DOI:** 10.1186/s12879-023-08859-9

**Published:** 2024-01-02

**Authors:** Eunmi Yang, Han Zo Choi, Subin Kim, Dong Hyun Oh, Mi Young Ahn, Sinyoung Ham, Eunyoung Lee, Jaehyun Jeon, Min-Kyung Kim, Hee-Chang Jang, Sang-Won Park, Jae-Phil Choi

**Affiliations:** 1 Division of Infectious Disease, Seoul Medical Center, 156, Sinnae-Ro, Jungnang-Gu, Seoul, 05505 Republic of Korea; 2 Department of Emergency Medicine, KyungHee University Hospital at Gangdong, Seoul, South Korea; 3 Seoul Veterans Hospital Medical Center, Seoul, Korea; 4 Department of Internal Medicine, Seoul National University Boramae Medical Center, 20 Boramae-Ro 5-Gil, Dongjak-Gu, Seoul, 07061 Korea; 5 Division of Infectious Diseases, Department of Internal Medicine, National Medical Center, Seoul, Korea; 6 National Institute of Infectious Disease, Korea National Institute of Health, Korea Disease Control and Prevention Agency, Chungcheongbuk-Do, Cheongju-Si, Korea

**Keywords:** Remdesivir, COVID-19, Renal insufficiency, Propensity Score, Safety

## Abstract

**Backgrounds:**

Remdesivir (RDV) is an antiviral agent approved for the treatment of coronavirus disease 2019 (COVID-19); however, is not recommended for patients with renal impairment. Due to limitations associated with prospective clinical trials, real-world data on the safety and efficacy of RDV in patients with renal impairment are necessary.

**Methods:**

Propensity score-matched (PSM) retrospective analysis was conducted between March 2020 and September 2022 in COVID-19 patients with an eGFR < 30 mL/min in four Korean hospitals. The RDV treatment group was matched to the untreated control group. The safety and clinical outcomes in patients who received RDV were analyzed.

**Results:**

A total of 564 patients were enrolled; 229 patients received RDV either for treatment or prophylaxis. On day 5, no difference in nephrotoxicity was observed between the two groups, and liver enzyme levels were within the normal range. In multivariate analysis for new dialysis, RDV treatment was not a risk factor for new dialysis. Among the 564 patients, 417 were indicated for a 5-day course of RDV treatment and 211 patients were treated with RDV. After PSM, no differences in the clinical outcomes were observed between the two groups.

**Conclusion:**

RDV use in COVID-19 patients with renal impairment did not result in significant nephrotoxicity or hepatotoxicity.

**Supplementary Information:**

The online version contains supplementary material available at 10.1186/s12879-023-08859-9.

## Background

Coronavirus disease 2019 (COVID-19), caused by severe acute respiratory syndrome coronavirus 2 (SARS-CoV-2), was first identified in December 2019, with approximately 650 million confirmed cases and 6.6 million deaths worldwide [1]. Underlying medical conditions such as old age, obesity, diabetes mellitus, cerebrovascular disease, and chronic kidney disease are risk factors for severe COVID-19 [2].

Remdesivir (RDV) is a broad-spectrum antiviral agent approved for the treatment of COVID-19 [3, 4]. RDV has been shown to reduce recovery time in adults hospitalized with COVID-19 and to prevent disease progression in high-risk COVID-19 patients [5, 6].

On May 1^st^ 2020, the Food and Drug Administration (FDA) approved the use of RDV for the treatment of COVID-19; however, recommended against its use in patients with an estimated glomerular filtration rate (eGFR) < 30 mL/min per 1.72 m^2^. At present, the pharmacokinetics of RDV have not been well evaluated in patients with decreased renal function. In addition, RDV formulations contain excipient sulfobutylether-beta-cyclodextrin (SBECD), which is cleared renally and accumulates in patients with renal impairment [3].

Recent studies containing a small number of participants have shown that RDV administration in patients with renal impairment is safe and is not associated with serious adverse effects [7, 8]. It was also reported that there was no clinically significant accumulation of RDV or its metabolites in patients with end-stage renal disease (ESRD) on hemodialysis [9]. This study aimed to provide information regarding the safety and efficacy of RDV in patients with renal impairment during the COVID-19 pandemic in Korea.

## Methods

### Study population and design

A multicenter retrospective cohort study of patients with laboratory-confirmed SARS-CoV-2 infection was conducted in Seoul, Korea. Four general hospitals designated for the treatment of patients with COVID-19 participated in this study. Patients admitted to hospitals between March 1^st^, 2020 and September 30^th^, 2022 were enrolled. Each case of SARS-CoV-2 infection was confirmed using reverse transcription polymerase chain reaction (RT-PCR). The inclusion criteria were as follows: (1) adult (≥ 19 years old) patients and (2) eGFR < 30 ml/min per 1.73 m^2^ prior to the first dose of RDV administration. Exclusion criteria included elevated alanine aminotransferase (ALT) level > 5 times the upper limit of the normal range (ULN) and confirmed SARS-CoV-2 infection three days after hospitalization. During the study period, the Delta and Omicron variants were dominant in Korea from July 2021 to December 2021 and February 2022 to July 2022, respectively.

### Criteria for RDV treatment

Remdesivir was administered in a 3-day course to prevent disease progression or in a 5-day course for treatment according to the Korea Disease Control and Prevention Agency (KDCA) guidelines [10]. The 3-day protocol for RDV administration required the following: (1) aged ≥ 60 years or aged ≥ 12 years with underlying disease (chronic respiratory disease, hypertension, cardiovascular disease, cerebrovascular disease, diabetes mellitus, body mass index (BMI) ≥ 30 kg/m^2^, immunosuppressed condition, chronic renal disease, chronic liver disease, active cancer, or sickle cell disease) and (2) symptom onset within 7 days and no requirement of oxygen supplement. The 5-day protocol for RDV administration required the following: (1) oxygen saturation of less than or equal to 94% for room air, (2) requirement for oxygen supplementation, or (3) chest imaging suggestive of viral pneumonia. All patients received symptomatic and standard care including oxygen, baricitinib, tocilizumab, and dexamethasone, regardless of whether RDV was administered.

### Data collection and study outcomes

Baseline characteristics, underlying disease, oxygen status, laboratory tests, patient management, and clinical outcomes data were collected from electronic medical records. Patients were divided into two groups for data analysis: a RDV-treated group and a control group that did not receive RDV. The Charlson comorbidity index was used to score the severity of comorbid conditions [11]. The modified World Health Organization (WHO) ordinal scale and National Early Warning Score-2 (NEWS-2) were used to evaluate disease severity [12, 13]. The modified ordinal scale was as follows: (1) no limitation of daily activities); (2) limitation of daily activities but no need for supplemental O_2_; (3) need for supplemental O_2_ via nasal prong; (4) need for supplemental O_2_ via facial mask; (5) need for high-flow supplemental O_2_ or noninvasive mechanical ventilation; (6) need for invasive mechanical ventilation; (7) multi-organ failure or the need for extracorporeal membrane oxygenation therapy; (8) death.

The primary outcome was the safety of RDV compared to the eGFR, creatinine, aspartate transaminase (AST), and ALT levels 5 days after the initiation of RDV treatment. New hemodialysis in patients not receiving dialysis at baseline during hospitalization was also compared between the two groups. The secondary outcomes were oxygen requirement during hospitalization, aggravation of disease severity according to the modified ordinal scale, and mortality in patients who received a 5-day course of RDV for therapeutic purposes.

### Statistical analysis

Continuous variables were presented as medians and interquartile ranges. Categorical variables were presented as numbers and percentages. The patients were divided into two groups (RDV-treated group and standard care group). To compare the two groups, the Mann–Whitney U-test was used for continuous variables, and the chi-square test or Fisher’s exact test were used for categorical variables. To eliminate the effect of confounding variables that influence outcome variables, when analyzing basic characteristics, the propensity score matching (PSM) method was used to collect data in both groups. For PSM analysis, missing data in rows were excluded. Patients receiving RDV were matched 1:1 with standard care patients according to the propensity score using exact matching. Using matched data, differences between the RDV-treated group and standard care group outcome variables were analyzed again. If significant variables were found when comparing the matched data of both groups, multivariate logistic regression analysis was performed with these significant variables.

For subgroup analysis, patients who required 5-day course of RDV treatment were selected from the total patient population. This subgroup was further divided into RDV- and standard-treatment groups. Fifty patients were randomly selected from the RDV group because two groups were similar in size. When comparing the basic characteristics of the 50 randomly selected individuals in the population, no statistical differences were found (Supplementary Tables S1 and S2). Patients using RDV who were randomly selected were matched 1:1 with standard care patients according to the propensity score using exact matching.

All statistical analyses were performed using R software version 4.1.2 (R Foundation for Statistical Computing, Vienna, Austria). *P*-values were based on a two-sided significance level of 0.05.

## Results

### Baseline characteristics

**Fig. 1:**
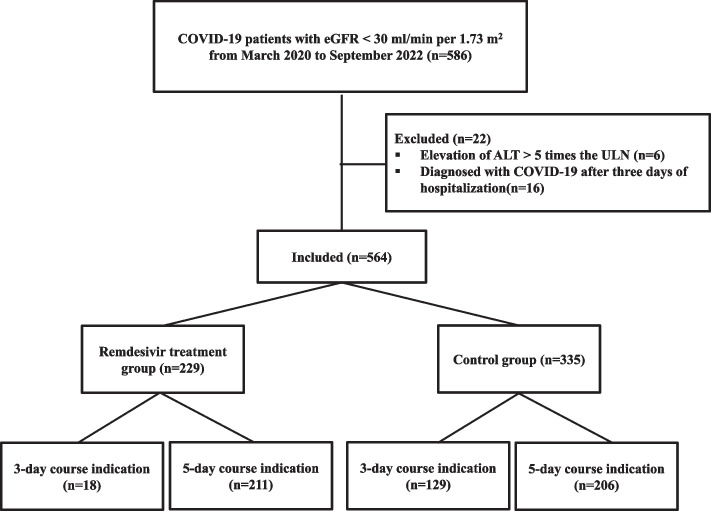
Flow chart representing the COVID-19 patients in the study. Abbreviations: COVID-19, Coronavirus Disease-19; eGFR, estimated glomerular filtration rate; AST, aspartate aminotransferase; ULN, upper limit of the normal range

A total of 586 patients were included in the cohort. Six patients were excluded due to elevated ALT levels, and 16 patients were excluded because they were diagnosed with COVID-19 after three days of hospitalization. A total of 564 patients were enrolled, 229 (40.6%) of whom received RDV (Fig. 1). The median duration of RDV treatment was 5 days (interquartile range (IQR), 3–5 days). Remdesivir administration was discontinued early in 39 patients, all of whom were in the 5-day course RDV treatment group. Of the 39 patients, eight were discontinued due to suspected complications: one patient had elevated liver enzyme levels and seven patients had deteriorated kidney function (Supplementary Table S3). All patients were included in the analysis, regardless of the duration of RDV treatment or whether treatment was discontinued or not. Supplementary Table S4 shows the concomitant drugs administered to patients during the study period.

Table 1 presents the baseline characteristics of the RDV-treated and control groups before and after matching. Prior to matching, substantial differences were observed between the two groups. Most factors that differed between the two groups were used for PS matching. Supplementary Table S5 shows the factors. The patients who received RDV were more likely to have hypertension, diabetes mellitus, or higher Charlson comorbidity scores. This group was also more likely to have a higher NEWS-2 score at admission, a higher modified ordinal scale score, higher risk of pneumonia, higher initial AST and ALT levels, a higher instance of steroid use, and increased oxygen requirements. After PS matching, the patients’ baseline characteristics were well-balanced between the two groups; however, the presenting disease severities, such as NEWS-2 score, modified ordinal scale, presence of pneumonia, and oxygen requirement, were still significantly higher in the RDV treatment group than in the control group.

**Table 1:** Baseline characteristics of patients with an eGFR of less than 30 mL/min/1.73m^2^

	Unmatched cohort			Propensity score-matched cohort		
	Remdesivir (*N* = 229)	Standard care (*N* = 335)	*P* value	Remdesivir (*N* = 178)	Standard care (*N* = 178)	*P* value
Age (year), median (IQR)	76 (66–83)	73 (60–83)	0.026	75 (66–83)	74 (63.8–84)	0.405
Male, N (%)	75 (32.8)	147 (43.9)	0.009	54 (30.3)	64 (36.0)	0.260
BMI (kg/m^2^), median (IQR)	23.3 (20.8–26.4)	22.8 (20.4–25.5)	0.205	23.3 (20.8–26.4)	23 (20.2–24.9)	0.242
Underlying disease, N (%)						
Hypertension	194 (84.7)	256 (76.4)	0.016	152 (85.4)	139 (78.1)	0.075
Diabetes mellitus	142 (62.0)	170 (50.7)	0.008	112 (62.9)	105(59.0)	0.447
Congestive heart disease	19 (8.3)	40 (11.9)	0.165	15 (8.4)	24 (13.5)	0.127
Cerebrovascular accident	42 (18.3)	41 (12.2)	0.045	36 (20.2)	28 (15.7)	0.270
Chronic liver disease	14 (6.1)	15 (4.5)	0.388	13 (7.3)	10 (5.6)	0.518
Solid cancer	30 (13.1)	33 (9.9)	0.229	22 (12.4)	16 (9.0)	0.303
Hematologic malignancy	3 (1.3)	1 (0.3)	0.309	1 (0.6)	1 (0.6)	0.999
ESRD (iHD or PD)	70 (30.6)	109 (32.5)	0.622	61 (34.3)	52 (29.2)	0.305
Kidney transplantation	8 (3.5)	15 (4.5)	0.562	5 (2.8)	7 (3.9)	0.557
Immunosuppressant use, N (%)	12 (5.2)	18 (5.4)	0.945	8 (4.5)	10 (5.6)	0.629
Charlson Comorbidity Index, score, median (IQR)	7 (5–8)	6 (4–8)	0.012	7 (5–8)	7 (5–8)	0.431
Baseline severity						
NEWS-2 score at admission, median (IQR)	5 (2–8)	1 (0–4)	< 0.001	5 (2–8)	2 (1–5)	< 0.001
Disease severity scores, median (IQR)	3 (2–3)	2 (1–2)	< 0.001	3 (1.8–3)	2 (1–3)	< 0.001
Pneumonia, N (%)	172/225 (76.4)	169/324 (52.2)	< 0.001	137 (77)	100 (56.2)	< 0.001
Remdesivir 5-day course indication^a^	211 (92.1)	206 (61.5)	< 0.001	161 (90.4)	123 (69.1)	< 0.001
Steroid use for treatment^b^	171 (74.7)	83 (24.8)	< 0.001	85 (47.8)	81 (45.5)	0.671
Oxygen requirement, N (%)	127 (55.5)	71 (21.2)	< 0.001	100 (56.2)	47 (26.4)	< 0.001
No oxygen requirement	102 (44.5)	264 (78.8)	< 0.001	78 (43.8)	131 (73.6)	< 0.001
Nasal cannula	102 (44.5)	46 (13.7)		81 (45.5)	33 (18.5)	
Facial mask	11 (4.8)	10 (3.0)		7 (3.9)	6 (3.4)	
High flow nasal cannula	8 (3.5)	11 (3.3)		7 (3.9)	5 (2.8)	
Invasive ventilation	6 (2.6)	4 (1.2)		5 (2.8)	3 (1.7)	
ECMO	0 (0)	0 (0)		0 (0)	0 (0)	
Initial laboratory result, median (IQR)						
Creatinine (mg/dL)	3.2 (2.4–6.7)	3.6 (2.5–7.8)	0.168	3.5 (2–8)	3 (3–7)	0.631
AST (IU/L)	34 (22–53)	24 (17–35)	< 0.001	32.5 (21–51)	24 (17–37)	< 0.001
ALT (IU/L)	17 (12–27)	15 (11–22)	0.006	17 (12–27)	16 (11–22.3)	0.089
eGFR (mL/min/1.73m^2^)	18 (8–24)	15 (6–23)	0.058	16 (7–23.3)	16.5 (7–23)	0.822

*Abbreviations*: *eGFR* estimated glomerular filtration rate, *IQR* interquartile range, *BMI* body mass index (weight in kilograms divided by height in meters squared), *ESRD* end-stage renal disease, *iHD* intermittent hemodialysis, *PD* peritoneal dialysis, *NEWS-2* National Early Warning Score-2, *ECMO* extracorporeal membrane oxygenation, *AST* aspartate aminotransferase, *ALT* alanine aminotransferase

^a^Indications: (1) oxygen saturation of less than or equal to 94% for room air, (2) requirement for oxygen supplementation, or (3) chest imaging suggestive of viral pneumonia

^b^Steroid use for COVID-19 treatment

### Primary outcomes

The safety indicators for RDV treatment are presented in Table 2. On day 5, creatinine decreased by 0.52 mg/dL in the treatment group and by 0.45 mg/dL in the control group. eGFR increased in both groups (2.1 mL/min/1.73 m^2^ and 1 mL/min/1.73 m^2^, respectively). After PS matching, the creatinine levels and eGFR were not significantly different between the two groups. AST and ALT levels were higher in the treatment group, but within the normal range. Patients in the RDV treatment group started hemodialysis more frequently than those in the standard group (*P* = 0.034). To determine whether RDV treatment was independently associated with new dialysis, we performed a multivariate logistic regression analysis of the two groups after adjusting for confounding factors (NEWS-2 score, pneumonia, oxygen requirement, AST, and RDV treatment). In the multivariate analysis of new dialysis, RDV treatment was not a risk factor for new dialysis (Table 3).

**Table 2:** Safety indicators of remdesivir treatment for patients with an eGFR of less than 30 mL/min/1.73m^2^

	Unmatched cohort			Propensity score-matched cohort		
	Remdesivir (*N* = 229)	Standard care (*N* = 335)	*P* value	Remdesivir (*N* = 178)	Standard care (*N* = 178)	*P* value
Day 5 laboratory result, median (IQR)						
Creatinine (mg/dL)	2.7 (1.7–7.5)	3.2 (1.9–7.6)	0.084	2.9 (1.7–7.7)	3 (1.9–6.7)	0.328
AST (IU/L)	27 (18–40)	22 (16–32)	0.003	27 (18–39)	23 (16–33)	0.037
ALT (IU/L)	19.5 (13–31.25)	16 (11–24)	0.001	19 (13–29.3)	15 (10.8–26)	0.017
eGFR (mL/min/1.73m^2^)	21.4 (6.85–37)	16 (6–31)	0.032	20.7 (6.1–37.1)	18 (7–31)	0.275
New dialysis in those not receiving dialysis at baseline	14 (6.1)	6 (1.8)	0.009	14 (7.9)	5 (2.8)	0.034

**Table 3:** Multivariate logistic regression of new dialysis in those not receiving dialysis at baseline with propensity score matching data

	Odds ratio	Confidence interval	*P* value
NEWS-2 score at admission (increasing 1 score)	1.090	0.936–1.268	0.269
Pneumonia (vs. no pneumonia)	2.620	0.565–12.158	0.219
Oxygen requirement (vs. no oxygen requirement)	0.878	0.218–3.537	0.855
AST (increasing 1 IU/L)	1.004	0.997–1.012	0.278
Remdesivir (vs standard care)	2.491	0.833–7.455	0.103

We evaluated baseline non-dialysis patients individually to exclude the effect of baseline dialysis on outcomes among study patients. No significant differences were observed in safety outcomes between the two groups, and RDV administration was not associated with new onset dialysis (Supplementary Tables 6–8).

### Baseline characteristics of patients who received a 5-day course of RDV treatment

A total of 417 patients were indicated for a 5-day course of RDV treatment, of which 211 (50.7%) were treated with RDV (Table 4). Prior to matching, patients in the treatment group were more likely to have hypertension, diabetes mellitus, steroid use, and oxygen requirement and exhibited higher NEWS-2 scores, disease severity scores, and AST levels. All factors that showed differences in the two groups were used in PS matching. Supplementary Table S5 shows the factors. After PS matching, the patient characteristics and disease severity were well balanced between the two groups; however, the steroid use in the RDV treatment group was higher than that in the control group (80% vs. 42%, *P* < 0.001).

**Table 4:** Baseline characteristics of patients with an eGFR of less than 30 mL/min/1.73m^2^ (5-day course of remdesivir treatment)

	Unmatched cohort			Propensity score-matched cohort		
	Remdesivir (*N* = 211)	Standard care (*N* = 206)	*P* value	Remdesivir (*N* = 50)	Standard care (*N* = 50)	*P* value
Age (year), median (IQR)	76 (67–83)	77.5 (64–85)	0.794	79 (71–85.3)	79 (64.8–85)	0.661
Male, N (%)	70 (33.2)	91 (44.2)	0.021	18 (36)	21 (42)	0.539
BMI (kg/m^2^), median (IQR)	23.3 (20.6–26.6)	22.6 (20.2–25)	0.119	22.4 (19.7–26.9)	22.5 (19.7–25.9)	0.924
Underlying disease, N (%)						
Hypertension	180 (85.3)	150 (72.8)	0.002	47 (94)	41 (82)	0.065
Diabetes mellitus	133 (63.0)	104 (50.5)	0.01	27 (54)	27 (54)	0.999
Congestive heart disease	18 (8.5)	27 (13.1)	0.132	6 (12)	13 (26)	0.074
Cerebrovascular accident	38 (18.0)	29 (14.1)	0.274	8 (16)	7 (14)	0.779
Chronic liver disease	12 (5.7)	8 (3.9)	0.389	1 (2)	2 (4)	0.999
Solid cancer	25 (11.8)	19 (9.2)	0.383	6 (12)	2 (4)	0.269
Hematologic malignancy	3 (1.4)	1 (0.5)	0.623	0 (0)	0 (0)	
ESRD (iHD or PD)	61 (28.9)	56 (27.2)	0.695	21 (42)	14 (28)	0.142
Kidney transplantation	8 (3.8)	9 (4.4)	0.766	0 (0)	1 (2)	0.999
Immunosuppressant use, N (%)	12 (5.7)	10 (4.9)	0.704	1 (2)	0 (0)	0.999
Steroid use for treatment^a^	171 (81.0)	77 (37.4)	< 0.001	40 (80.0)	21 (42.0)	< 0.001
Charlson Comorbidity Index, score, median (IQR)	7 (5–8)	7 (5–8)	0.499	7 (5–9)	7 (6–8)	0.473
Baseline severity						
NEWS-2 score at admission, median (IQR)	5 (2–8)	2 (1–7)	< 0.001	4 (1.8–7)	4 (1–8.3)	0.895
Disease severity scores, median (IQR)	3 (2–3)	2 (1–3)	< 0.001	3 (2–4)	2.5 (1–3.3)	0.338
Pneumonia, N (%)	172/207 (83.1)	169/204 (82.8)	0.947	41 (82)	35 (70)	0.160
Oxygen requirement, N (%)	127 (60.2)	71 (34.5)	< 0.001	30 (60)	25 (50)	0.315
No oxygen requirement	84 (39.8)	135 (65.5)	< 0.001	20 (40)	25 (50)	0.294
Nasal cannula	102 (48.3)	46 (22.3)		21 (42)	14 (28)	
Facial mask	11 (5.2)	10 (4.9)		5 (10)	4 (8)	
High flow nasal cannula	8 (3.8)	11 (5.3)		1 (2)	5 (10)	
Invasive ventilation	6 (2.8)	4 (1.9)		3 (6)	2 (4)	
ECMO	0 (0)	0 (0)		0 (0)	0 (0)	
Initial laboratory result, median (IQR)						
Creatinine (mg/dL)	3.2 (2.4–6)	3.1 (2.4–6.8)	0.864	3 (2.8–7.3)	3 (2–7.3)	0.532
AST (IU/L)	35 (23.3–53.8)	26 (18–41.5)	< 0.001	32 (21.8–47.3)	27.5 (18–41.8)	0.187
ALT (IU/L)	18 (13–28)	16 (11–24)	0.039	16 (11.8–25.3)	16 (12–23.3)	0.733
eGFR (mL/min/1.73m^2^)	18 (8–24)	18 (7–24)	0.436	16.5 (7.8–22)	18.5 (7–24.3)	0.392

^a^Steroid use for COVID-19 treatment

### Secondary outcomes (Clinical outcomes of patients who received a 5-day course of RDV treatment)

Prior to matching, more patients in the RDV treatment group required an oxygen supply during hospitalization (87.7% vs. 55.3%, *P* < 0.001) and the duration of hospitalization was longer than that in the control group (12 days vs. 10 days, *P* = 0.034). There were no significant differences in mortality or ordinal severity scores on day 21 or at discharge (Table 5). After PS matching, there were no significant differences between the two groups in terms of the oxygen requirement, disease severity score, duration of hospitalization, or mortality.

**Table 5:** Clinical outcomes of patients with an eGFR of less than 30 mL/min/1.73m^2^ (5-day course of remdesivir treatment)

	Unmatched cohort			Propensity score-matched cohort		
	Remdesivir (*N* = 211)	Standard care (*N* = 206)	*P* value	Remdesivir (*N* = 50)	Standard care (*N* = 50)	*P* value
Day 5 laboratory result, median (IQR)						
Creatinine (mg/dL)	2.6 (1.7–6.6)	2.9 (1.8–6.3)	0.431	3.1 (1.7–7.4)	2.9 (1.7–6)	0.817
AST (IU/L)	28 (18–41.5)	24 (18–35)	0.141	27 (17–37.5)	23 (16.5–33.5)	0.574
ALT (IU/L)	20 (13–33)	16 (11–28)	0.025	18 (11.5–23)	16 (12–24.5)	0.957
eGFR (mL/min/1.73m^2^)	22 (7–37.5)	19.5 (7–33)	0.233	18.8 (6.8–35.3)	18 (7–34.3)	0.839
New dialysis in those not receiving dialysis at baseline	13 (6.2)	5 (2.4)	0.061	0 (0)	1 (2)	0.999
Oxygen requirement during hospitalization, N (%)	185 (87.7)	114 (55.3)	< 0.001	41 (82)	35 (70)	0.160
No oxygen requirement	26 (12.3)	92 (44.7)	< 0.001	9 (18)	15 (30)	0.049
Nasal cannula	131 (62.1)	57 (27.7)		28 (56)	16 (32)	
Facial mask	8 (3.8)	11 (5.3)		2 (4)	3 (6)	
High flow nasal cannula	35 (16.6)	33 (16.0)		5 (10)	13 (26)	
Invasive ventilation	11 (5.2)	12 (5.8)		6 (12)	3 (6)	
ECMO	0 (0)	1 (0.5)		0 (0)	0 (0)	
Progression of oxygen supply^a^	83 (39.3)	64 (31.1)	0.077	16 (32)	17 (34)	0.832
Median time to progression of oxygen supply, days (IQR)	1 (1–2)	1 (1–3)	0.619	1 (1–2)	1 (1–2)	0.688
Disease severity scores on ordinal scale						
Progression of ordinal score during hospitalization	0 (-2–2)	0 (0–1)	0.071	1 (-3–2)	1 (0–2)	0.602
Ordinal score at day 21 or discharge, median (IQR)	2 (1–6)	1 (1–2)	0.159	2 (1–8)	1 (1–2)	0.221
Hospitalization						
Median duration of hospitalization (IQR)	12 (7–19)	10 (7–16)	0.034	11 (8–17.3)	11.5 (8–17.5)	0.994
Median duration of hospitalization among those who did not die or transfer (IQR)	11 (8–16.5)	11 (8–14)	0.321	10 (8–12)	11 (8–16.5)	0.097
Mortality^b^	46/191 (24.1)	42/197 (21.3)	0.516	13/48 (27.1)	6/47 (12.8)	0.081
Mortality through day 21	37/191 (19.4)	36/197 (18.3)	0.782	10/48 (20.8)	5/47 (10.6)	0.173
COVID-19 attributable mortality through day 21	33/191 (17.3)	31/197 (15.7)	0.683	8/48 (16.7)	4/47 (8.5)	0.232
Mortality through day 28	41/191 (21.5)	40/197 (20.3)	0.778	11/48 (22.9)	6/47 (12.8)	0.197
COVID-19 attributable mortality through day 28	37/191 (19.4)	34/197 (17.3)	0.590	9/48 (18.8)	5/47 (10.6)	0.265

*Abbreviation*: *COVID-19* Coronavirus Disease-19

^a^Progression of oxygenation methods without oxygen supply, nasal prong, face mask, high-flow nasal cannula, invasive mechanical ventilation, or extracorporeal membrane oxygenation

^b^All-cause mortality during hospitalization

An additional analysis of all-cause mortality was conducted to analyze the effect of steroid administration on the outcomes. Multivariate logistic regression analysis of all-cause mortality during hospitalization, with steroid use, RDV use, and disease severity score at admission as confounding factors, indicated that steroid and RDV use were not independent risk factors for all-cause mortality (Table 6).

**Table 6:** Multivariate logistic regression of all-cause mortality during hospitalization in patients

	Odds ratio	Confidence interval	*P* value
Steroid use for treatment (vs. no steroid use)	3.080	0.707–13.421	0.134
Remdesivir (vs. standard care)	0.302	0.084–1.081	0.066
Disease severity scores at admission (1 score increase)	2.702	1.708–4.276	< 0.001

## Discussion

In this retrospective cohort study, patients with an eGFR less than 30 mL/min/1.73 m^2^ who received RDV had no aggravation of the creatinine levels and eGFR on day 5 and did not differ from those of the control group. On day 5, AST and ALT levels were within the normal range. In addition, RDV treatment was not an independent risk factor for new dialysis treatments during hospitalization. In patients indicated for a 5-day course of RDV, there were no differences between the two groups in terms of the disease severity score at day 21, oxygen requirement during hospitalization, or mortality.

There have been concerns regarding the safety of RDV use in SARS-CoV-2 infected patients with renal impairment due to the accumulation of SBECD. Recent studies have reported conflicting results regarding RDV treatment in these patients [14, 15]. Our study aimed to confirm the safety of RDV in patients with renal impairment using safety indicators such as the AST, ALT, and creatinine levels, as well as eGFR and new hemodialysis in the Korean population. Overall, patients with a severe clinical status or underlying diseases tended to receive RDV. We used PS matching to balance the baseline demographic characteristics between the two groups; however, disease severity indicators, such as NEWS-2 score, modified ordinal scale, presence of pneumonia, and oxygen requirement, could not be balanced. Although patients who received RDV had more severe diseases, creatinine levels and eGFR improved on day 5, and the liver function test results were within the normal range. There were also no differences in patients who initiated new hemodialysis between the RDV and standard groups after correcting for confounding factors. Our study showed that RDV treatment in patients with renal impairment was safe and well-tolerated.

Our study tried to reflect real-world situations of renal impairment by evaluating dialysis and non-dialysis patients together. However, dialysis may change the pharmacokinetics of RDV. Therefore, baseline non-dialysis patients were evaluated individually. No difference was observed in liver enzymes, creatinine, and eGFR on day 5 between the two groups. Additionally, RDV was not a risk factor for new-onset dialysis. It showed the RDV treatment is safe in patients with non-dialysis renal impairment.

Even among patients who were indicated for a 5-day course of RDV, those with a more severe clinical status and underlying disease were more likely to receive RDV than those in the control group. Nevertheless, the 21-day disease severity score and mortality rate were not higher in the RDV-treated group than in the control group. There was also no difference in the oxygen requirement or duration of hospitalization between the groups after PS matching. Steroid use for COVID-19 treatment was higher in the RDV-treated group than in the control group despite PS matching. We assumed that the reason for increased steroid use in the RDV-treated group was that those patients had a more severe disease status and there were uncorrected confounding factors. Nevertheless, there was no difference in the outcomes, and it is presumed that RDV is beneficial for the treatment of COVID-19 in patients with renal impairment. To analyze the effect of RDV and steroid use on mortality in patients with renal impairment, a multivariate analysis of all-cause mortality was performed. In the multivariate logistic regression analysis, neither RDV treatment nor steroid use significantly improved the survival of COVID-19 patients with renal impairment.

Our study had several limitations. First, this was a multicenter retrospective study, and the decision to administer RDV was made by the clinicians. Therefore, there could be biases in each center’s policies and clinicians’ preferences. Second, steroid use was not corrected using PS matching in the 5-day course of RDV treatment indication study. Furthermore, there may be unadjusted confounding factors such as the severity of pneumonia. Steroid use may also have affected the secondary outcomes. Additionally, concomitant drugs may affect clinical outcomes. However, we believe these drugs are unlikely to affect outcomes because of the few patients. Further studies are required to determine the effects of steroid use, concomitant drugs with RDV, and appropriate COVID-19 treatment in patients with renal impairment.

## Conclusion

In conclusion, the use of RDV for COVID-19 in patients with renal impairment was confirmed to be safe. Furthermore, RDV was not significantly associated with hepatotoxicity or renal toxicity. In patients with severe conditions, such as pneumonia and oxygen requirement, the administration of RDV did not seem to significantly improve mortality; therefore, better-designed studies are necessary.

### Supplementary Information

**Additional file 1:** **Supplementary Table S1.** Baseline characteristics of patients with an eGFR of less than 30 mL/min/1.73m^2^ between the population and sample groups. **Supplementary Table S2.** Clinical outcomes of patients with an eGFR of less than 30 mL/min/1.73m^2^ between the population and sample groups. **Supplementary Table S3.** Reasons for discontinuation of remdesivir treatment in 39 patients. **Supplementary Table S4.** Concomitant drugs administrated to study patients. **Supplementary Table S5.** Factors used in propensity score matching. **Supplementary Table S6.** Baseline characteristics of non-dialysis patients with an eGFR of less than 30 mL/min/1.73m^2^. **Supplementary Table S7.** Safety indicators of remdesivir treatment for non-dialysis patients with an eGFR of less than 30 mL/min/1.73m^2^. **Supplementary Table S8.** Multivariate logistic regression of new dialysis in non-dialysis patients using propensity score matching data.

## Data Availability

The datasets used and/or analysed during the current study available from the corresponding author on reasonable request.

## Abbreviations

COVID-19: Coronavirus disease 2019
SARS-CoV-2: Severe acute respiratory syndrome coronavirus 2
RDV: Remdesivir
FDA: Food and Drug Administration
eGFR: Estimated glomerular filtration rate
SBECD: Sulfobutylether-beta-cyclodextrin
ESRD: End-stage renal disease
RT-PCR: Reverse transcription polymerase chain reaction
ALT: Alanine aminotransferase
ULN: Upper limit of the normal range
KDCA: Korea Disease Control and Prevention Agency
BMI: Body mass index
WHO: World Health Organization
NEWS-2: National Early Warning Score-2
PSM: Propensity score matching
IQR: Interquartile range
