# Supplementary material for: Propensity score matched analysis for the safety and effectiveness of remdesivir in COVID-19 patients with renal impairment
Source: BMC Infect Dis. 2024 Jan 2;24:3. doi: 10.1186/s12879-023-08859-9 (PMC10759744; doi:10.1186/s12879-023-08859-9)
Supplement: Supplementary file 1 — Additional file 1: Supplementary Table S1. Baseline characteristics of patients with an eGFR of less than 30 mL/min/1.73m2 between the population and sample groups. Supplementary Table S2. Clinical outcomes of patients with an eGFR of less than 30 mL/min/1.73m2 between the population and sample groups. Supplementary Table S3. Reasons for discontinuation of remdesivir treatment in 39 patients. Supplementary Table S4. Concomitant drugs administrated to study patients. Supplementary Table S5. Factors used in propensity score matching. Supplementary Table S6. Baseline characteristics of non-dialysis patients with an eGFR of less than 30 mL/min/1.73m2. Supplementary Table S7. Safety indicators of remdesivir treatment for non-dialysis patients with an eGFR of less than 30 mL/min/1.73m2. Supplementary Table S8. Multivariate logistic regression of new dialysis in non-dialysis patients using propensity score matching data. [file 12879_2023_8859_MOESM1_ESM.docx]

**Supplementary Table S1. Baseline characteristics of patients with an eGFR of less than 30 mL/min/1.73m^2^ between the population and sample groups**

|  | Remdesivir  (N=211) | Remdesivir  random sample  (N=50) | *P* value |
| --- | --- | --- | --- |
| Age (year), median (IQR) | 76 (67-83) | 79 (71-85.3) | 0.159 |
| Male, N (%) | 70 (33.2) | 18 (36) | 0.704 |
| BMI (kg/m^2^), median (IQR) | 23.3 (20.6-26.6) | 22.4 (19.7-26.9) | 0.418 |
| Underlying disease, N (%) |  |  |  |
| Hypertension | 180 (85.3) | 47 (94) | 0.101 |
| Diabetes mellitus | 133 (63.0) | 27 (54) | 0.238 |
| Congestive heart disease | 18 (8.5) | 6 (12) | 0.422 |
| Cerebrovascular accident | 38 (18.0) | 8 (16) | 0.737 |
| Chronic liver disease | 12 (5.7) | 1 (2) | 0.473 |
| Solid cancer | 25 (11.8) | 6 (12) | 0.976 |
| Hematologic malignancy | 3 (1.4) | 0 (0) | 0.999 |
| ESRD (iHD or PD) | 61 (28.9) | 21 (42) | 0.073 |
| Kidney transplantation | 8 (3.8) | 0 (0) | 0.360 |
| Immunosuppressant use, N (%) | 12 (5.7) | 1 (2) | 0.473 |
| Steroid use for treatment^*^ | 174 (82.5) | 42 (84) | 0.796 |
| Charlson’s Comorbidity Index, score, median (IQR) | 7 (5-8) | 7 (5-9) | 0.255 |
| Baseline severity |  |  |  |
| NEWS-2 score at admission, median (IQR) | 5 (2-8) | 4 (1.8-7) | 0.255 |
| Disease severity scores, median (IQR) | 3 (2-3) | 3 (2-4) | 0.67 |
| Pneumonia, N (%) | 172/207 (83.1) | 41 (82) | 0.854 |
| Oxygen requirement, N (%) | 127 (60.2) | 30 (60) | 0.98 |
| No oxygen requirement | 84 (39.8) | 20 (40) |  |
| Nasal cannula | 102 (48.3) | 21 (42) |  |
| Facial mask | 11 (5.2) | 5 (10) |  |
| High flow nasal cannula | 8 (3.8) | 1 (2) |  |
| Invasive ventilation | 6 (2.8) | 3 (6) |  |
| ECMO | 0 (0) | 0 (0) |  |
| Initial laboratory result, median (IQR) |  |  |  |
| Creatinine (mg/dL) | 3.2 (2.4-6) | 3 (2.8-7.3) | 0.482 |
| AST (IU/L) | 35 (23.3-53.8) | 32 (21.8-47.3) | 0.542 |
| ALT (IU/L) | 18 (13-28) | 16 (11.8-25.3) | 0.416 |
| eGFR (mL/min/1.73m^2^) | 18 (8-24) | 16.5 (7.8-22) | 0.204 |

Abbreviations: eGFR, estimated glomerular filtration rate; IQR, interquartile range; BMI, body mass index (weight in kilograms divided by height in meters squared); ESRD, end-stage renal disease; iHD, intermittent hemodialysis; PD, peritoneal dialysis; NEWS-2, [National Early Warning Score](http://www.rcplondon.ac.uk/projects/outputs/national-early-warning-score-news-2)-2[; ECMO, extracorporeal membrane oxygenation; AST, aspartate aminotransferase; ALT, alanine aminotransferase.](http://www.rcplondon.ac.uk/projects/outputs/national-early-warning-score-news-2)

* Steroid use for COVID-19 treatment.

**Supplementary Table S2. Clinical outcomes of patients with an eGFR of less than 30 mL/min/1.73m^2^ between the population and sample groups**

|  | Remdesivir  (N=211) | Remdesivir  random sample  (N=50) | *P* value |
| --- | --- | --- | --- |
| Day 5 laboratory result, median (IQR) |  |  |  |
| Creatinine (mg/dL) | 2.6 (1.7-6.6) | 3.1 (1.7-7.4) | 0.573 |
| AST (IU/L) | 28 (18-41.5) | 27 (17-37.5) | 0.344 |
| ALT (IU/L) | 20 (13-33) | 18 (11.5-23) | 0.134 |
| eGFR (mL/min/1.73m^2^) | 22 (7-37.5) | 18.8 (6.8-35.3) | 0.373 |
| New dialysis in those not receiving dialysis at baseline | 13 (6.2) | 0 (0) | 0.138 |
| Oxygen requirement during hospitalization, N (%) | 185 (87.7) | 41 (82) | 0.288 |
| No oxygen requirement | 26 (12.3) | 9 (18) |  |
| Nasal cannula | 131 (62.1) | 28 (56) |  |
| Facial mask | 8 (3.8) | 2 (4) |  |
| High flow nasal cannula | 35 (16.6) | 5 (10) |  |
| Invasive ventilation | 11 (5.2) | 6 (12) |  |
| ECMO | 0 (0) | 0 (0) |  |
| Progression of oxygen supply^*^ | 83 (39.3) | 16 (32) | 0.336 |
| Median time to progression of oxygen supply, days (IQR) | 1 (1-2) | 1 (1-2) | 0.932 |
| Disease severity scores on ordinal scale |  |  |  |
| Progression of ordinal score during hospitalization | 0 (-2-2) | 1 (-3-2) | 0.762 |
| Ordinal score at day 21 or discharge, median (IQR) | 2 (1-6) | 2 (1-8) | 0.850 |
| Hospitalization |  |  |  |
| Median duration of hospitalization (IQR) | 12 (7-19) | 11 (8-17.3) | 0.356 |
| Median duration of hospitalization among those who did not die or transfer (IQR) | 11 (8-16.5) | 10 (8-12) | 0.949 |
| Mortality^**^ | 46/191 (24.1) | 13/48 (27.1) | 0.667 |
| Mortality through day 21 | 37/191 (19.4) | 10/48 (20.8) | 0.82 |
| COVID-19 attributable mortality through day 21 | 33/191 (17.3) | 8/48 (16.7) | 0.92 |
| Mortality through day 28 | 41/191 (21.5) | 11/48 (22.9) | 0.828 |
| COVID-19 attributable mortality through day 28 | 37/191 (19.4) | 9/48 (18.8) | 0.922 |

*Progression of oxygenation methods without oxygen supply, nasal prong, face mask, high-flow nasal cannula, invasive mechanical ventilation, or extracorporeal membrane oxygenation.

**All-cause mortality during hospitalization.

**Supplementary Table S3. Reasons for discontinuation of remdesivir treatment in 39 patients**

| Reason | No. (%) |
| --- | --- |
| Elevated liver enzyme levels | 1 (2.56) |
| Deterioration of kidney function | 7 (17.95) |
| Transfer to other hospitals | 11 (28.21) |
| Death | 6 (15.38) |
| Concerns regarding low eGFR | 4 (10.26) |
| Headache | 1 (2.56) |
| Difficult to continue intravenous catheter | 1 (2.56) |
| Unknown cause | 8 (20.51) |

**Supplementary Table S4. Concomitant drugs administrated to study patients**

|  | Remdesivir | Standard care |
| --- | --- | --- |
| Nirmatrelvir and ritonavir (Paxlovid) | 1^*^ | 26 |
| Molnupiravir | 0 | 1 |
| Monoclonal antibody (Regdanvimab) | 7 | 21 |
| Tocilizumab | 1 | 0 |
| Baricitinib | 6 | 6 |

*Administered only 1 day before remdesivir treatment

**Supplementary Table S5. Factors used in propensity score matching**

| **Table 1^*^** | Age, male, hypertension, diabetes mellitus, cerebrovascular accident, the Charlson Comorbidity Index, NEWS-2 score at admission, disease severity scores, pneumonia, steroid use for treatment, oxygen requirement, AST, ALT |
| --- | --- |
| **Table 4** | Male, hypertension, diabetes mellitus, steroid use for treatment, NEWS-2 score at admission, disease severity scores, oxygen requirement, AST, ALT |

^*^The remdesivir 5-day indication was removed from PS matching because it was associated with disease severity scores.

**Supplementary Table S6. Baseline characteristics of non-dialysis patients with an eGFR of less than 30 mL/min/1.73m^2^**

|  | Unmatched cohort | | | Propensity score-matched cohort | | |
| --- | --- | --- | --- | --- | --- | --- |
|  | Remdesivir  (N=159) | Standard care  (N=226) | *P* value | Remdesivir  (N=117) | Standard care  (N=117) | *P* value |
| Age (year), median (IQR) | 79 (69-85) | 77.5 (65.0-84.3) | 0.092 | 79 (71-85) | 78 (66.5-85) | 0.317 |
| Male, N (%) | 57 (35.8) | 107 (47.3) | 0.025 | 79 (67.5) | 68 (58.1) | 0.137 |
| BMI (kg/m^2^), median (IQR) | 23.5 (20.8-26.9) | 22.7 (20.5-25.4) | 0.111 | 23.5 (20.8-26.9) | 23 (20.3-25.0) | 0.151 |
| Underlying disease, N (%) |  |  |  |  |  |  |
| Hypertension | 138 (86.8) | 162 (71.7) | <0.001 | 104 (88.9) | 97 (82.9) | 0.189 |
| Diabetes mellitus | 96 (60.4) | 110 (48.7) | 0.023 | 72 (61.5) | 69 (58.1) | 0.594 |
| Congestive heart disease | 11 (6.9) | 26 (11.5) | 0.133 | 7 (6.0) | 17 (14.5) | 0.031 |
| Cerebrovascular accident | 32 (20.1) | 33 (14.6) | 0.154 | 27 (23.1) | 21 (17.9) | 0.331 |
| Chronic liver disease | 12 (7.5) | 7 (3.1) | 0.047 | 11 (9.4) | 4 (3.4) | 0.062 |
| Solid cancer | 23 (14.5) | 29 (12.8) | 0.644 | 16 (13.7) | 10 (8.5) | 0.212 |
| Hematologic malignancy | 3 (1.9) | 1 (0.4) | 0.311 | 1 (0.9) | 0 (0) | 0.999 |
| Kidney transplantation | 6 (3.8) | 12 (5.3) | 0.482 | 3 (2.6) | 5 (4.3) | 0.722 |
| Immunosuppressant use, N (%) | 9 (5.7) | 15 (6.6) | 0.696 | 5 (4.3) | 7 (6.0) | 0.553 |
| Charlson Comorbidity Index, score, median (IQR) | 7 (5-8) | 7 (5-8) | 0.202 | 7 (5-8) | 7 (5-8) | 0.438 |
| Baseline severity |  |  |  |  |  |  |
| NEWS-2 score at admission, median (IQR) | 5 (2-9) | 2 (1-5) | <0.001 | 5 (2.5-8) | 2 (1-7) | <0.001 |
| Disease severity scores, median (IQR) | 3 (2-3) | 2 (1-3) | <0.001 | 3 (2-4) | 2 (1-3) | <0.001 |
| Pneumonia, N (%) | 126 (80.8) | 126 (57.8) | <0.001 | 97 (82.9) | 82 (70.1) | 0.021 |
| Steroid use for treatment^**^ | 127 (79.9) | 64 (28.3) | <0.001 | 68 (58.1) | 62 (53.0) | 0.430 |
| Oxygen requirement, N (%) | 99 (62.3) | 59 (26.1) | <0.001 | 76 (65.0) | 41 (35.0) | <0.001 |
| No oxygen requirement | 60 (37.7) | 167 (73.9) | <0.001 | 41 (35.0) | 76 (65.0) | <0.001 |
| Nasal cannula | 77 (48.4) | 39 (17.3) |  | 58 (49.6) | 29 (24.8) |  |
| Facial mask | 10 (6.3) | 10 (4.4) |  | 7 (6.0) | 6 (5.1) |  |
| High flow nasal cannula | 7 (4.4) | 9 (4.0) |  | 6 (5.1) | 5 (4.3) |  |
| Invasive ventilation | 5 (3.1) | 1 (0.4) |  | 5 (4.3) | 1 (0.9) |  |
| ECMO | 0 (0) | 0 (0) |  | 0 (0) | 0 (0) |  |
| Initial laboratory result, median (IQR) |  |  |  |  |  |  |
| Creatinine (mg/dL) | 2.6 (2.2-3.5) | 2.7 (2.3-3.8) | 0.575 | 3.5 (2.3-4.4) | 3.4 (2.2-4.3) | 0.893 |
| AST (IU/L) | 37 (25-58) | 26 (19-39) | <0.001 | 36 (24-58) | 27 (19-40) | <0.001 |
| ALT (IU/L) | 18 (13-30) | 15 (11-23) | 0.014 | 19 (13.0-30.5) | 16 (11-23) | 0.053 |
| eGFR (mL/min/1.73 m^2^) | 21.5 (15-26) | 21 (14.2-25.0) | 0.296 | 22 (15-26) | 23 (16.5-26.0) | 0.486 |

Abbreviations: eGFR, estimated glomerular filtration rate; IQR, interquartile range; BMI, body mass index (weight in kilograms divided by height in meters squared); ESRD, end-stage renal disease; iHD, intermittent hemodialysis; PD, peritoneal dialysis; NEWS-2, [National Early Warning Score](http://www.rcplondon.ac.uk/projects/outputs/national-early-warning-score-news-2)-2[; ECMO, extracorporeal membrane oxygenation; AST, aspartate aminotransferase; ALT, alanine aminotransferase.](http://www.rcplondon.ac.uk/projects/outputs/national-early-warning-score-news-2)

* Indications: (1) oxygen saturation of less than or equal to 94% for room air, (2) requirement for oxygen supplementation, or (3) chest imaging suggestive of viral pneumonia.

** Steroid use for COVID-19 treatment.

**Supplementary Table S7. Safety indicators of remdesivir treatment for non-dialysis patients with an eGFR of less than 30 mL/min/1.73m^2^**

|  | Unmatched cohort | | | Propensity score-matched cohort | | |
| --- | --- | --- | --- | --- | --- | --- |
|  | Remdesivir  (N=159) | Standard care  (N=226) | *P* value | Remdesivir  (N=117) | Standard care  (N=117) | *P* value |
| Day 5 laboratory result, median (IQR) |  |  |  |  |  |  |
| Creatinine (mg/dL) | 2 (1.5-3.1) | 2.3 (1.6-3.3) | 0.181 | 1.9 (1.5-3.1) | 2.3 (1.6-3.1) | 0.432 |
| AST (IU/L) | 28.5 (20-45) | 24 (17-33) | 0.007 | 28 (19.0-39.5) | 24 (18.0-37.5) | 0.184 |
| ALT (IU/L) | 21 (14-34) | 16 (11-28) | 0.004 | 21 (14.0-33.5) | 17 (12-29) | 0.129 |
| eGFR (mL/min/1.73 m^2^) | 30 (18.6-42.2) | 25 (16-36) | 0.074 | 30 (17.0-43.3) | 26 (17.8-37.0) | 0.325 |
| New dialysis in those not receiving dialysis at baseline | 14 (8.8) | 6 (2.7) | 0.007 | 14 (12.0) | 5 (2.6) | 0.006 |

**Supplementary Table S8. Multivariate logistic regression of new dialysis in non-dialysis patients using propensity score matching data**

|  | Odds ratio | Confidence interval | *P* value |
| --- | --- | --- | --- |
| NEWS-2 score at admission (increasing 1 score) | 1.052 | 0.905-1.223 | 0.509 |
| Pneumonia (vs. no pneumonia) | 2.119 | 0.445-10.096 | 0.346 |
| Oxygen requirement (vs. no oxygen requirement) | 0.912 | 0.227-3.658 | 0.896 |
| AST (increasing 1 IU/L) | 1.003 | 0.995-1.011 | 0.478 |
| Remdesivir (vs standard care) | 2.47 | 0.805-7.58 | 0.114 |
